# Establishing Normal Serum Values of Neurofilament Light Chains and Glial Fibrillary Acidic Protein Considering the Effects of Age and Other Demographic Factors in Healthy Adults

**DOI:** 10.3390/ijms25147808

**Published:** 2024-07-17

**Authors:** Alexander Rodero-Romero, Enric Monreal, Raquel Sainz-Amo, José Manuel García Domínguez, Noelia Villarrubia, Jose Luís Veiga-González, José Ignacio Fernández-Velasco, Haydee Goicochea-Briceño, Fernando Rodríguez-Jorge, Susana Sainz de la Maza, Juan Luís Chico-García, Alfonso Muriel, Jaime Masjuan, Lucienne Costa-Frossard, Luisa María Villar

**Affiliations:** 1 Department of Immunology, Hospital Universitario Ramón y Cajal, Red Española de Esclerosis Múltiple (REEM), Red de Enfermedades Inflamatorias (REI), ISCIII, Instituto Ramón y Cajal de Investigación Sanitaria, 28034 Madrid, Spain; alexander.rodero@salud.madrid.org (A.R.-R.); noelia.villarrubia@salud.madrid.org (N.V.); joseluis.veiga@salud.madrid.org (J.L.V.-G.); jfvelasco@salud.madrid.org (J.I.F.-V.); 2 Department of Neurology, Hospital Universitario Ramón y Cajal, Red Española de Esclerosis Múltiple (REEM), Red de Enfermedades Inflamatorias (REI), ISCIII, Instituto Ramón y Cajal de Investigación Sanitaria, 28034 Madrid, Spain; penry02@hotmail.com (E.M.); raquelsainzamo@gmail.com (R.S.-A.); deterodriguez@hotmail.com (F.R.-J.); susana.sainzdelamaza@salud.madrid.org (S.S.d.l.M.); juanluis.chico.garcia@gmail.com (J.L.C.-G.); jaime.masjuan@salud.madrid.org (J.M.); lucienne.costa@salud.madrid.org (L.C.-F.); 3 Department of Neurology, Hospital General Universitario Gregorio Marañón, 28007 Madrid, Spain; jgarciadominguez@salud.madrid.org (J.M.G.D.);; 4 Department of Biostatistics, Hospital Universitario Ramón y Cajal, CIBERESP, Instituto Ramón y Cajal de Investigación Sanitaria, 28034 Madrid, Spain; alfonso.muriel@uah.es

**Keywords:** neurodegeneration, neuroinflammation, biomarkers, healthy controls

## Abstract

Multiple studies have shown the importance of blood-based biomarkers indicating axonal damage (serum neurofilament light chains [sNfL]) or astroglia activation (serum glial fibrillary acidic protein [sGFAP]) for monitoring different neurological diseases. However, normal values of these variables remain to be clearly defined, partly due to the influence of different demographic factors. We investigated demographic differences in a cohort of healthy volunteers. A cross-sectional study was conducted including 116 healthy controls with ages between 18 and 69 years (67.5% females; n = 79). sNfL and sGFAP concentrations were measured using single-molecule arrays. Age and body mass index affected sNfL values, and age was found to be the most important factor. The normal values changed with age, and we established normal values for individuals younger than 45 years as <10 pg/mL and for controls older than 45 years as <15 pg/mL. We established normal values at <10 pg/mL for individuals younger than 45 years and <15 pg/mL for older individuals. Alternatively, a Z-score of 1.5 was relevant for all controls. sGFAP was only affected by age. Differences in normal values were evident by 55 years. The highest normality limit for sGFAP was 140 pg/mL for controls under 55 years and 280 for older controls. We defined normal levels for sNfL and sGFAP and their corresponding age-associated changes. These data may contribute to the application of such variables in clinical practice.

## 1. Introduction

Blood-based biomarkers in neurological diseases have recently gained considerable attention because of their accessibility and minimally invasive procedures. Neurofila-ments and glial fibrillary acidic protein (GFAP) have emerged as promising candidates for examining disease pathophysiology in neurological conditions, such as multiple sclerosis (MS), Alzheimer disease, Parkinson’s disease, and others [1,2,3,4,5].

Neurofilaments are structural axonal cytoskeleton proteins composed of three subunits—(1) heavy, (2) intermediate, and (3) light chains—based on their molecular weights. These chains constitute polymers located under the plasma membrane. After axonal damage occurs, polymers become disorganized and are released into the extracellular space medium, as occurs in certain neurological diseases [6,7,8]. The most commonly detected subunit is the neurofilament light chain (NfL). Its levels were first evaluated in cerebrospinal fluid (CSF) [6,9]. However, the onset of a highly sensitive technique, the single-molecule array (SIMOA), allowed researchers to quantify NfL levels in serum (sNfL) [7,8,9]. A good correlation exists between NfL levels in CSF and serum, thus enabling the use of sNfL as a reliable biomarker of axonal damage [9].

The potential use of this biomarker has been observed in various neurological diseases. High levels of sNfL correlate with MS activity, predicting relapses, disease progression, and increases in disability along the disease course [4,8,10,11]. Recent research has demonstrated that elevated sNfL levels can also predict future brain volume loss in MS patients, highlighting its importance in long-term disease monitoring [12]. Additionally, elevated sNfL levels are linked with the presence of amyloid plaques and tau in Alzheimer’s disease, making it a useful tool for early diagnosis and monitoring of disease progression [5,13]. sNfL levels are also known to predict cognitive decline and brain atrophy in Alzheimer’s disease [13,14]. In Parkinson’s disease, sNfL levels rise as the disease worsens, correlating with declining motor skills and cognitive abilities [15,16]. Moreover, sNfL can help to distinguish Parkinson’s from similar conditions like multiple system atrophy (MSA) and progressive supranuclear palsy (PSP), as sNfL levels are much higher in MSA and PSP [16]. Overall, sNfL is a promising biomarker for various neurodegenerative and neuroinflammatory diseases, providing valuable insights into disease mechanisms and contributing to establishing personalized treatments. However, its normal values in healthy individuals are not fully defined, limiting the applicability of this biomarker in clinical practice. The normal values of sNfL have been difficult to determine due to its correlation with variables such as body mass index (BMI) and age [4,13,17,18,19,20]. Statistical tools, such as the Z-score, are used to standardize sNfL levels by comparing them to a reference group and adjusting for age and BMI [19,20]. However, despite this advancement, the Z-score values are not fully established in clinical practice, since their calculations remain cumbersome. Additionally, the interval of normality for this score remains to be clearly established.

GFAP, which is predominantly expressed in astrocytes, began to emerge as a biomarker for astrogliosis and CNS injury [5,21]. The serum values of sGFAP can be quantified by using highly sensitive techniques, such as SIMOA and the i-STAT TBI plasma assay. Elevated sGFAP levels correlate with micro-damage in normal-appearing white and grey matter, thus suggesting its involvement in the pathophysiology of different neurological diseases [5,21,22,23]. In MS, increased GFAP levels correlate with disease severity and progression, providing insights into the extent of astrocytic involvement [10,22,23]. In Alzheimer’s disease, high GFAP levels are linked to amyloid plaque load and neurofibrillary tangles, suggesting its potential in early diagnosis and disease monitoring [21,24]. Elevated GFAP levels correlate with cognitive decline and brain atrophy, providing a non-invasive method to track disease progression [5,21]. Similarly, in Parkinson’s disease, higher GFAP levels are associated with more severe motor dysfunction and cognitive decline, making it a potential marker for disease severity [5,25].

This growing body of research highlights the importance of GFAP in understanding and managing neurodegenerative and neuroautoimmune diseases, enhancing diagnostic accuracy and patient care. Since GFAP is a relatively novel biomarker, normal values are still under investigation. Currently, it has been observed that GFAP levels are associated with an individual’s age [5,24,25,26], but while efforts are being made to establish a Z-score for this biomarker, results have not yet been published [5,24].

Despite the promising role of sNfL and sGFAP as biomarkers in neurodegenerative and neuroautoimmune diseases, a significant gap remains in terms of establishing their normal values in healthy individuals. A few studies use healthy controls to compare these biomarkers with patient cohorts, but they often do so without defining standardized normal ranges [27,28,29]. This limitation hinders the clinical applicability of sNfL and sGFAP, making it difficult to interpret elevated levels accurately.

This study aims to establish the normal values of sNfL and sGFAP in healthy individuals, examining the influence of putative confounding factors such as age, sex, and BMI on their levels.

## 2. Results

### 2.1. Serum Neurofilaments (sNfL)

#### 2.1.1. Association with Sex

We explored differences in sNfL values among the 79 females (67.5%) and 38 males (32.5%) participating in the study. We did not find significant differences between sexes for sNFL. The median values for sNfL were 6.3 pg/mL, and the 25–75% interquartile range (25–75% IQR) was 4.3–9.8 pg/mL for males. These values were 7.20 pg/mL and 4.7–9.8 pg/mL for females (*p* = 0.35).

Then, to gain a comprehensive understanding of how sex, age, sGFAP, and BMI collectively and individually impact the sNfL values, and to avoid misinterpretation of the association of different factors with sNfL levels due to the interference of other variables, a logistic regression was also conducted (Table 1). The variance inflation factor (VIF) scores of these variables ranged from 1.05 to 1.16, indicating no multicollinearity between them. No association with sex was found using this method either.

#### 2.1.2. Association with BMI

**Figure 1:**
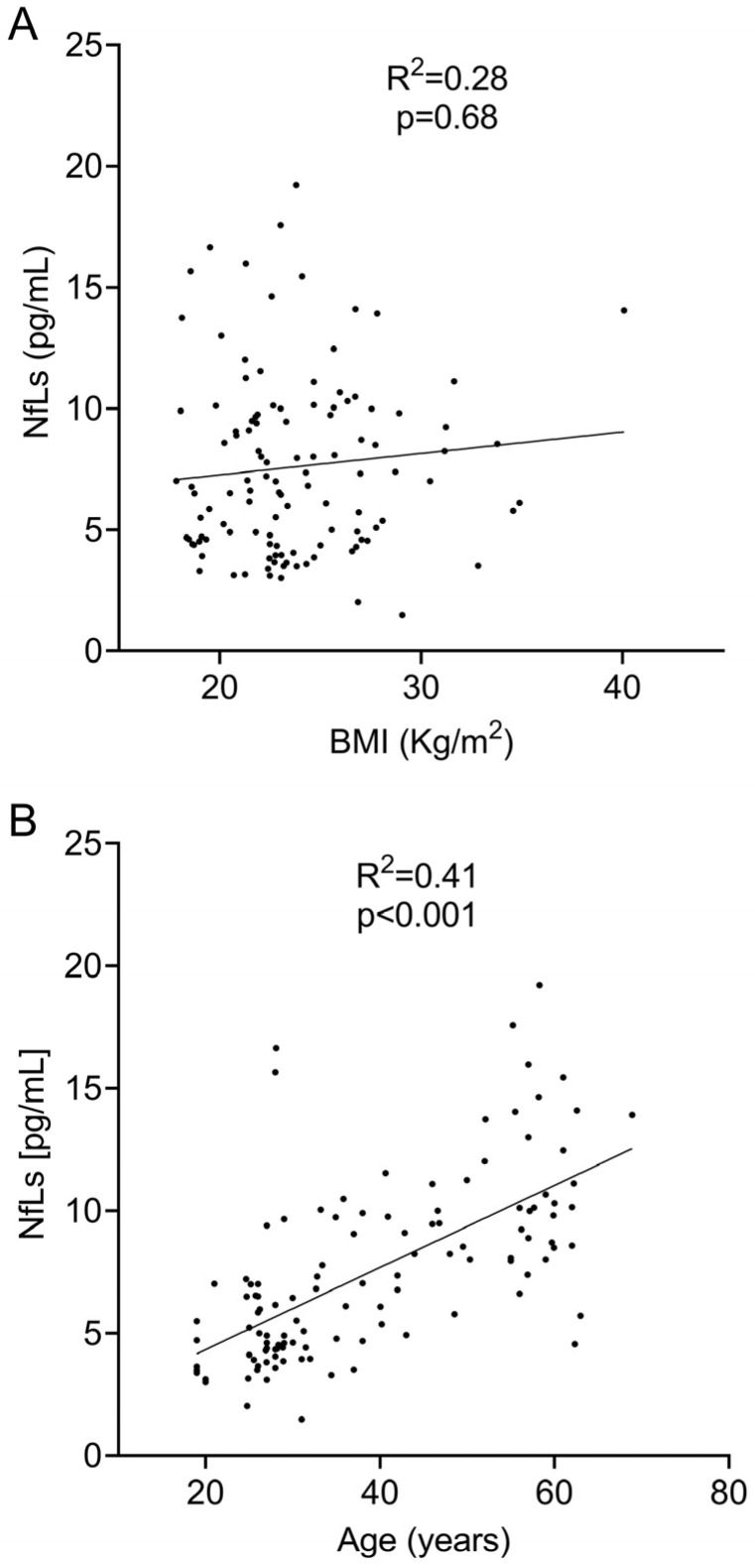
Linear regression analysis between sNfL levels and BMI (**A**) and age (**B**).

We next explored the association of body mass index and sNfL values. We did not find any significant correlation when we evaluated them based on linear regression (R^2^ = 0.28; *p* = 0.68, Figure 1A) and Spearman’s (r = 0.06; *p* = 0.50) tests. However, the logistic regression showed a negative correlation between sNfL levels and BMI. For each point of BMI increase, sNfL values decreased by −0.15 pg/mL (Table 1).

#### 2.1.3. Association with Age

To assess the relationship between sNfL levels and age, we first analyzed the linear regression between both variables. A positive correlation was found between sNfL and age (R^2^ = 0.41, *p* < 0.0001, Figure 1B). The correlation analysis using Spearman’s test revealed an even stronger positive correlation (r = 0.67; *p* < 0.0001). Finally, the logistic regression analysis also showed a significant correlation between sNFL levels and age (Table 1). This analysis indicated that sNfL levels increased by 0.16 pg/mL for every year of age.

**Figure 2:**
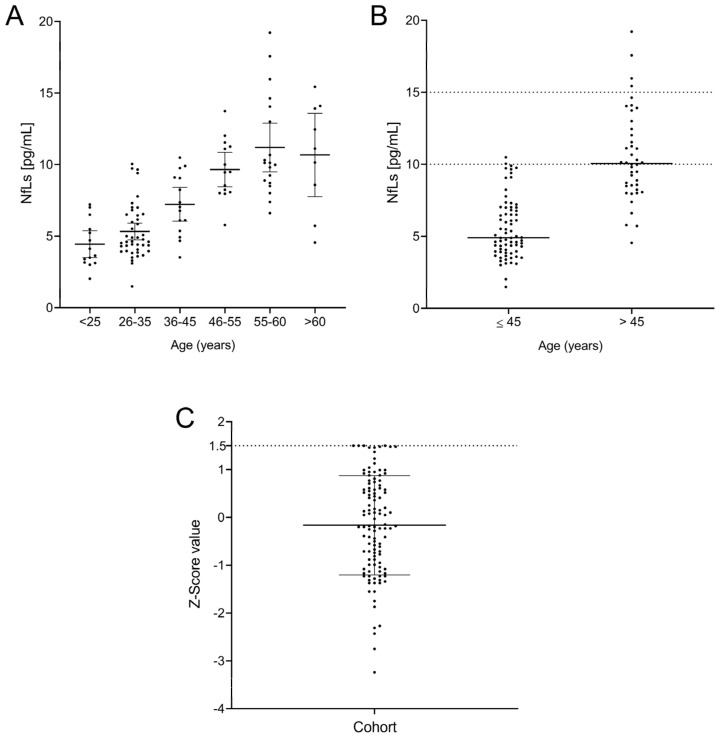
Levels of neurofilaments by age group (**A**), divided into individuals younger and older than 45 years, with their normal values as the 90th percentile (**B**) and Z-score (**C**) for the entire cohort.

We then explored sNfL values in controls, which were classified according to the following age ranges: (1) ≤25 years, (2) 26–35 years, (3) 36–45 years, (4) 46–55 years, (5) 56–60 years, and (6) >60 years (Figure 2A). No significant differences in sNfL values were found between the first three groups. However, participants older than 45 years showed clearly higher values. Accordingly, we classified patients in two groups, those with ages ≤ 45 years and those with ages > 45. We found clear differences in sNfL values between them (*p* < 0.0001, Figure 2B). No group showed a normal distribution based on the Kolmogorov–Smirnov test (*p* = 0.003 for Group ≤ 45 and *p* = 0.008 for Group > 45), and thus, we calculated the median values and 25–75% IQR for both groups. Additionally, we explored 90% percentiles, which are commonly used to establish normal values in healthy cohorts showing non-normal distribution [23]. Our results were 4.9 pg/mL (3.9–7.0; median [25%–75% IQR]), with a 90th percentile of 10 pg/mL for Group ≤ 45, and 10.0 pg/mL (IQR = 8.4–12.6), with a 90th percentile of 15 pg/mL for Group > 45. Only 1 of 72 controls belonging to the first group showed values higher than the 90th percentile (10 pg/mL). Accordingly, only 4 out of the 43 participants older than 45 years had sNfL values higher than 15 pg/mL. We then established that sNfL values lower than 10 pg/mL can be considered normal for individuals aged ≤ 45 years, and values lower than 15 pg/mL are the normal range in older individuals. Notably, all participants under 25 years had sNfL values lower than 7.5 pg/mL, so special consideration could be given to younger individuals with sNfL levels above these values.

We then examined the Z-scores in our cohort. All individuals showed values ≤ 1.5 (Figure 2C) independent of age. We then established that this value was the limit for healthy individuals.

### 2.2. Serum Glial Acidic Fibrillary Protein (sGFAP)

#### 2.2.1. Association with Sex

We next explored the sGFAP values in controls classified by their sex. We did not find significant differences within males, with a median value of 92.1 pg/mL [25–27% IQR: 73.19–119.6], or females, with a median of 101.3 pg/mL [25–75% IQR: 80.4–137], *p* = 0.25. A logistic regression analysis including BMI and age was also carried out (Table 2). It confirmed that sex does not affect the sGFAP levels. No multicollinearity was observed between these variables, with a VIF score close to a value of 1.

#### 2.2.2. Association with BMI

**Figure 3:**
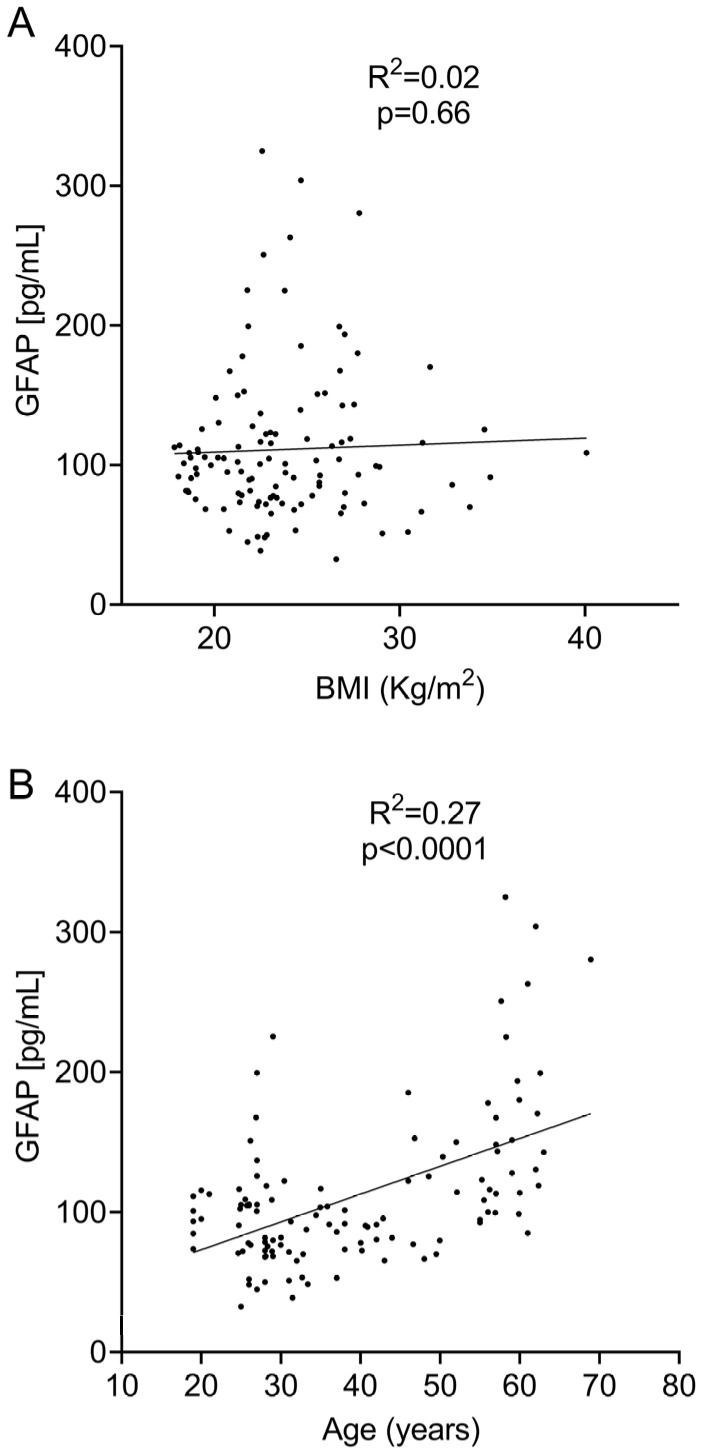
Linear regression analysis between sGFAP levels and BMI (**A**) and age (**B**).

Likewise, we did not find a significant association between sGFAP and BMI when they were analyzed using linear regression (R^2^ = 0.002; *p* = 0.66, Figure 3A) or a Spearman’s test (r = 0.03; *p* = 0.76). No association was obtained on the logistic regression analysis (Table 2).

#### 2.2.3. Association with Age

**Figure 4:**
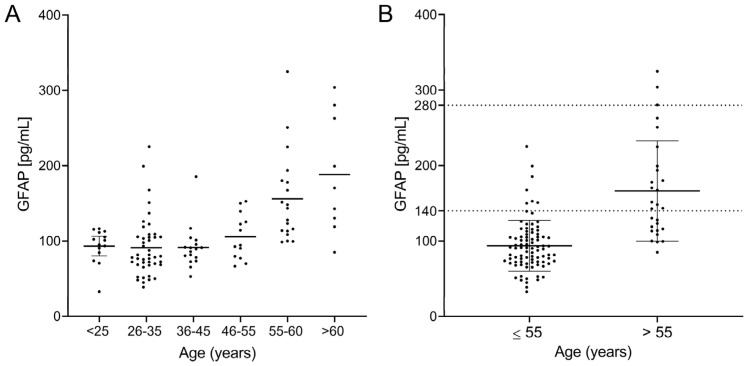
sGFAP levels according to age groups: (**A**) divided into individuals younger and older than 55 years with their normal values as the 90th percentile; (**B**) for the entire cohort.

We observed a clear correlation between sGFAP and age when they were analyzed using linear regression (R^2^ = 0.4110; *p* < 0.0001, Figure 3B), Spearman’s test (r = 0.42; *p* < 0.0001), and logistic regression (Table 2). This correlation indicates that the age range probably influences normal values. To explore this, we again divided the cohort into six different age groups. sGFAP values remained fairly stable in participants aged under 55 years and abruptly increased in controls older than 55 years (Figure 4A). Accordingly, we classified participants in two groups, those aged ≤ 55 years (Group ≤ 55) and older individuals (Group > 55). We again performed descriptive analyses and did not find a normal distribution in any group (*p* = 0.008 for Group ≤ 55 and *p* = 0.006 for Group > 55). The ≤55 group had a median value of 90.5 (25–75% IQR: 71.2–107.9) and a 90th percentile of 140 pg/mL. The >55 group showed a median value of 145.8 (25–75% IQR: 114.2–197.9) with a 90th percentile of 280 pg/mL (Figure 4B). We established values below the 90th percentile as the normal values for both age ranges. Only 6 of 88 controls in the ≤55 group showed values higher than the 90th percentile (140 pg/mL). Correspondingly, only 3 of 28 controls in the >55 group showed values higher than the 90th percentile (280 pg/mL). Notably, individuals under 25 years did not have particularly low sGFAP values.

## 3. Discussion

An increasing number of studies show that blood biomarkers, such as sNfl and sGFAP, are useful for the clinical assessment of patients with neurological diseases, including MS and Alzheimer’s disease [1,7,10,13,19]. However, blood biomarkers’ applicability remains limited, mainly because reference values are usually established according to specific studies’ patient populations, which challenges their widespread use in clinical settings. In addition, many studies use a single cut-off point for the entire cohort without considering important demographic factors, such as age, sex, and/or BMI [17,30,31,32]. However, it is crucial to establish normal values for these variables in a cohort of healthy individuals after considering demographic factors. This type of consideration would provide reference values for examining the value of these biomarkers under different clinical conditions.

Age is the primary confounding factor when examining serum biomarkers in neuro-degenerative diseases. Our results confirm previous findings that have determined that sNfL values increase with age [11,18,21,24]. In the case of sNfL, we observed that different normal values exist according to age. We observed that a cut-off value of 10 pg/mL, frequently used to differentiate low and high values of sNfL [8,11,19], only works for individuals ≤ 45 years. Even in this group, we noticed that individuals under 25 showed lower sNfL values, raising the question of whether separate normal values should be set for this age group in future studies to ensure appropriate usage. By contrast, for patients with ages > 45 years, the cut-off rose to 15 pg/mL. The positive correlation between sNfL and age corroborates that adjusting sNfL levels by age could improve the accuracy of this biomarker in clinical practice.

We also confirmed that BMI has a modest negative correlation with sNfL, although the effect of age was clearly higher. The cut-off values established in our work could be useful in clinical practice for rapid patient assessment. However, when more precise data are needed, the Z-score values, as previously described, could be the best option [20,31] The z-scores correct raw data by age and also by BMI values. It avoids overestimation of the values of SnfL in aged individuals, and may also avoid underestimation of sNfL data in patients with very high BMIs. We explored the normal values for the z-score. A value of 1.5 clearly defines healthy controls. Different studies have indicated that values between 1 and 2 can be good cut-off values for the z-score in MS [19,31]. Our data reinforce that the value of 1.5 can discriminate high from normal values. S. This may contribute to higher consistency and reproducibility of z-score values across different diseases.

sGFAP, a marker of astrogliosis/astrocyte activation, also represents a promising biomarker for predicting innate cell activation in different neurological diseases. However, normal values of this protein, in addition to the influence of demographic factors on normal values, have been far less studied [5,23,33,34,35]. We observed that only age had a significant influence on sGFAP levels. Unlike the gradual variations seen in sNfL across age ranges, sGFAP showed a more striking change at an age around 55 years. Below this age, the values remained stable across the studied ranges, while a clear increase occurred in individuals who were older than 55 years old. These data are interesting, since recent data suggest that GFAP plays a role in the immune senescence process [36,37]. Consequently, we established a cut-off value of 140 pg/mL for individuals younger than 55 years and 280 pg/mL for those who were older. Since the changes are very evident at 55 years of age, we believe that it may not be necessary to establish a reference Z-score for sGFAPs, since the normal levels established in the two age groups can be widely applied.

We describe now that the two cut-off values are essential for GFAP analyses. If a control cohort not separated by age is used, most healthy individuals should show high values at ages older than 55, and this would make the study of neurodegenerative diseases such as Alzheimer’s, where high sGFAP values have proven to be essential for the formation of Tau tangles, difficult [21]. Having age-stratified values should be very important in evaluating the impact of new treatments on the disease. The same is true for MS, where sGFAP, usually associated with older age, may play an important role in the progressive phase of the disease [23,24,25].

Our study has some limitations. The number of controls included was relatively low, and the data obtained should be validated in larger cohorts. Additionally, the study population may not be representative of the broader population in terms of age, sex, and BMI. In case of age, we wanted to cover an adult population ranging from 18 to 69 years, which may be useful for studying different neurological diseases in this age range. Future studies are warranted to explore the control values of pediatric patients and controls of older ages. Regarding sex, we were limited by donations, and a predominance of the female sex (67.5%) was observed in our cohort. However, as 32.5% males were included, we could clearly analyze the differences between sexes. The BMI value was not a prerequisite for inclusion in the study, and thus, it ranged between 17.85 and 40.08, which represents the general population in our environment, but does not reflect values in other settings.

Other limitations of the study were other potential confounding factors such as lifestyle, diet, physical activity, and genetic predispositions, which were not controlled. However, we asked the participants about toxic habits such as drug or alcohol intake, which could influence the results. They were included as exclusion criteria. Finally, all individuals included in the study were Caucasians living in Spain. We did not include other ethnic groups or geographic localizations. Further studies will demonstrate how these factors can influence sNfL and sGFAP values in a more general setting.

In summary, this work confirmed the influence of age and, to a lesser extent, BMI on the sNfL levels of healthy individuals. It describes the cut-off values of this variable depending on age. Additionally, we confirmed the importance of the Z-score to accurately determine the normal values of sNfL and established the best cut-off for this variable. On the other hand, we examined the normal values of sGFAP and determined their associations with age. The normal values defined for sNfL and sGFAP, considering age and demographic factors, enhance the accuracy of these biomarkers, enabling a more precise assessment of pathological conditions.

## 4. Materials and Methods

### 4.1. Study Design

This multi-center, cross-sectional study was approved by the Ethics Committee of the Ramón y Cajal University Hospital in Madrid (Ethics Committee approval number: 237–27). It was performed in the Hospital Universitario Ramón y Cajal and the Hospital General Universitario Gregorio Marañón, which shared the inclusion criteria and sample processing. All participants signed an informed consent form prior to entering the study.

### 4.2. Participants

A total of 116 healthy volunteers were enrolled in our study between August 2023 and February 2024. All participants were volunteers recruited among the personnel and students of both hospitals.

The inclusion criteria were age of 18 years or older, no evidence of disease, and absence of toxic habits (alcoholism or drug consumption).

The exclusion criteria were treatment with immunosuppressive drugs or chemotherapeutic agents.

A physician interviewed each control, although they did not conduct in-depth medical examinations.

Demographic information on every participant, including age, sex, and BMI (weight [kg]/height [m^2^]), was collected. The participants’ ages ranged from 18 to 69 years. The median age was 35 years [25–75% IQR: 27–55 years]. In total, 38 males (32.5%) and 79 females (67.5%) with a median BMI of 23.03 [25–75% IQR: 21.28–26.47] participated.

When needed, the participants were stratified by age into the following groups: (1) ≤25 years (n = 14), (2) 26–35 years (n = 43), (3) 36–45 years (n = 15), (4) 46–55 years (n = 14), (5) 56–60 years (n = 19), and (6) >65 years (n = 9).

### 4.3. Sample Processing

Serum samples were collected from the study participants by extracting 10 mL of blood into dry tubes. The tubes were kept for 30 min at room temperature to allow coagulation, then centrifuged at 3000× *g* for 5 min. After centrifugation, the serum was collected, aliquoted in 250 µL aliquots, and stored at −80 °C until analysis.

The concentrations of sNfL and sGFAP were assessed using the SIMOA NF-light™ Advantage Kit and the Simoa™ GFAP Discovery Kit, respectively (Quanterix, Billerica, MA, USA), on an SR-X instrument (Quanterix, Billerica, MA, USA) following the manufacturer’s instructions. SIMOA is an ultrasensitive immunoassay technology used to detect and quantify biomolecules at extremely low levels. The analysis followed a two-step assay protocol performed following the manufacturer’s instructions. In summary, paramagnetic beads coated alternatively with anti-NfL or anti-GFAP capture antibodies were incubated with the calibrators (undiluted), controls, and samples (diluted 1:4), always in duplicate, and with biotinylated detection antibodies. After incubation, the beads were washed and incubated with streptavidin-conjugated β-galactosidase. Then, they were washed again and incubated with the fluorimetric substrate. Finally, the plates were introduced into the SR-X instrument, where the microbeads were distributed into an array with 200.000 individual wells, each sized to hold a single microbead in a final volume of 50 fl. This small volume greatly increased the signal and the sensitivity of the detection. sNfl and sGFAP concentrations were automatically calculated using the standard curve.

This approach significantly increased the sensitivity and precision of the assay compared to traditional immunoassay techniques. The lower limits of quantification and detection were, respectively, 0.345 pg/mL and 0.085 pg/mL for the NF-L assay. For the GFAP test, they were 0.686 pg/mL and 0.211 and pg/mL, respectively.

### 4.4. Statistical Analysis

All statistical analyses were performed using GraphPad Prism 9.0 (GraphPad Prism Inc., San Diego, CA, USA) and Stata, version 14 (StataCorp LLC, Cambridge, MA, USA). All tests were two-tailed, and *p* <  0.05 was considered significant.

We performed descriptive analyses and expressed continuous variables as the median and 25–75% IQR values and assessed normality using the Kolmogorov–Smirnov test. We analyzed the association between demographic variables and sNfL or sGFAP levels by means of linear regression, Spearman correlation, and logistic regression tests.

Differences between groups were examined using the Mann–Whitney U and the Kruskal–Wallis multiple comparisons tests, along with Dunn’s post hoc test.

## 5. Conclusions

In this study, we established the normal values of sNfL and sGFAP in a cohort of healthy individuals and established their different associations with age. sNfL values increased gradually, thus reinforcing the usefulness of a z-score for their fine evaluation, at least for ages above 45 years. By contrast, sGFAP values remained stable for many years and increased abruptly at the age of 55 years. These data reinforce the utility of using two different normality values for evaluating this variable. When confirmed in larger cohorts, our results will improve the integration of these biomarkers in clinical protocols to improve the monitorization of different neurological conditions.

## Figures and Tables

**Table 1:** Results of the logistic regression analysis examining the associations between sNFL, sex, sGFAP, BMI, and age. The table displays the estimated coefficients, standard errors, *p*-values, and the 95% confidence interval for each predictor variable. sNFL: serum neurofilaments; sGFAP: serum glial acidic fibrillary protein; BMI: body mass index; SE: standard error.

sNFL	Coefficient	SE	*p*-Value	[95% Conf. Interval]	
sGFAP	0.010	0.005	0.077	−0.001	0.212
Age	0.162	0.023	0.000	0.116	0.208
BMI	−0.148	0.069	0.033	−0.285	−0.118
Sex	−0.393	0.555	0.994	−1.104	1.096

**Table 2:** Summary of logistic regression analysis assessing the associations of sGFAP with sex, sNfL, BMI, and age. The table includes the estimated coefficients, standard errors, *p*-values, and 95% CI for each predictor variable. BMI: body mass index; SE: standard error.

sGFAP	Coefficient	SE	*p*-Value	[95% Conf. Interval]	
sNfL	2.771	1.550	0.077	−0.300	5.843
Age	1.645	0.432	<0.001	0.788	2.501
BMI	−1.436	1.160	0.218	−3.736	0.862
Sex	0.479	9.798	0.959	−17.748	18.707

## Data Availability

The raw data can be obtained from the corresponding author upon reasonable request in the three years following the manuscript’s publication.
